# Supplementary material for: A New Graphical Method for Displaying Two-Dimensional Echocardiography Results in Dogs: Comprehensive Analysis of Results of Diagnostic Imaging Organized in a BOX (CARDIOBOX)
Source: Vet Sci. 2025 Jan 9;12(1):34. doi: 10.3390/vetsci12010034 (PMC11769013; doi:10.3390/vetsci12010034)
Supplement: Supplementary file 1 [file vetsci-12-00034-s001.zip › Figure S1-jotform english.pdf]

Basic Echocardiographic Interpretation

(translated from spanish: https://form.jotform.com/223278319852059)

Activity for veterinary specialists and clinicians.

Activity Introduction
We kindly ask you to watch this brief video where we will minimally explain the basic steps we will request from you.
https://youtu.be/U5pup4iB840

Anonymous Form - Basic Interpretation of Echocardiographic Reports
We will not ask for your name; you will only need to complete the data in this form with minimal private information of demographic interest. This exercise will take just a few minutes to complete.

- **How old are you?***
- **Where do you live?***
    - City
    - Country
- **What is your academic background?***
    - Veterinary student
    - Clinical veterinarian
    - Veterinary cardiologist
    - Veterinary anesthesiologist
    - Other veterinary specialty
    - I have no veterinary training

First Clinical Case
Interpret the following clinical case of a 5.5 kg poodle. It includes basic echocardiographic parameter values in the second column, and you must compare them with the reference values for its weight.

Medición	Caso "troy" caniche 5,5 kg	2.5-5 kg	5-10 kg	10-14 kg	14-17 kg	17-18 kg
**AI / Ao** (atrio aorta)	1,2	1 – 1,4	1 – 1,4	1 – 1,4	1 – 1,4	1 – 1,4
**IVSd** (septo en diástole)	6,4	4,8 -7,7	6,0 - 8,4	7,1 - 8,9	7,8 - 9,3	8,3 - 9,4
**LVDd** (cámara VI en diástole)	26,1	11,8 -25	20,2 - 28,9	25,3 - 31,7	28,7 -33,8	31,1 -34,4
**PLVWd** (pared libre VI en diástole)	6,7	3,75 - 6,8	4,8 - 6,8	5,7 - 7,2	6,3 - 7,5	6,7 - 7,6
**IVSs** (septo en sístole)	11,2	7,1 - 12,1	9,0 - 12,1	10,7 - 13,0	11,8 -13,6	12,5 -13,8
**LVDs** (cámara VI en sístole)	15,0	5,3 - 14,3	11,2 - 17,3	14,7 - 19,2	17,0 -20,6	18,7 -21,0
**PLVWs** (pared libre VI en sístole)	9,6	6,1 - 9,8	8,0 - 10,7	9,4 - 11,4	10,3 -11,9	10,9 -12,0

1a- **How would you describe the size of the LEFT ATRIUM?***

- Normal
- Increased
- Decreased

1b- **How would you describe the size of the INTERVENTRICULAR SEPTUM in DIASTOLE?***

- Normal
- Increased
- Decreased

1c- **How would you describe the size of the LEFT VENTRICULAR CHAMBER in DIASTOLE?***

- Normal
- Increased
- Decreased

1d- **How would you describe the size of the LEFT VENTRICULAR FREE WALL in DIASTOLE?***

- Normal
- Increased
- Decreased

1e- **How would you describe the size of the INTERVENTRICULAR SEPTUM in SYSTOLE?***

- Normal
- Increased

- Decreased

1f- **How would you describe the size of the LEFT VENTRICULAR CHAMBER in SYSTOLE?***

- Normal
- Increased
- Decreased

1g- **How would you describe the size of the LEFT VENTRICULAR FREE WALL in SYSTOLE?***

- Normal
- Increased
- Decreased

Second Clinical Case

Interpret the following clinical case of a 7 kg Maltese. Reference ranges are provided in the graphical charts.

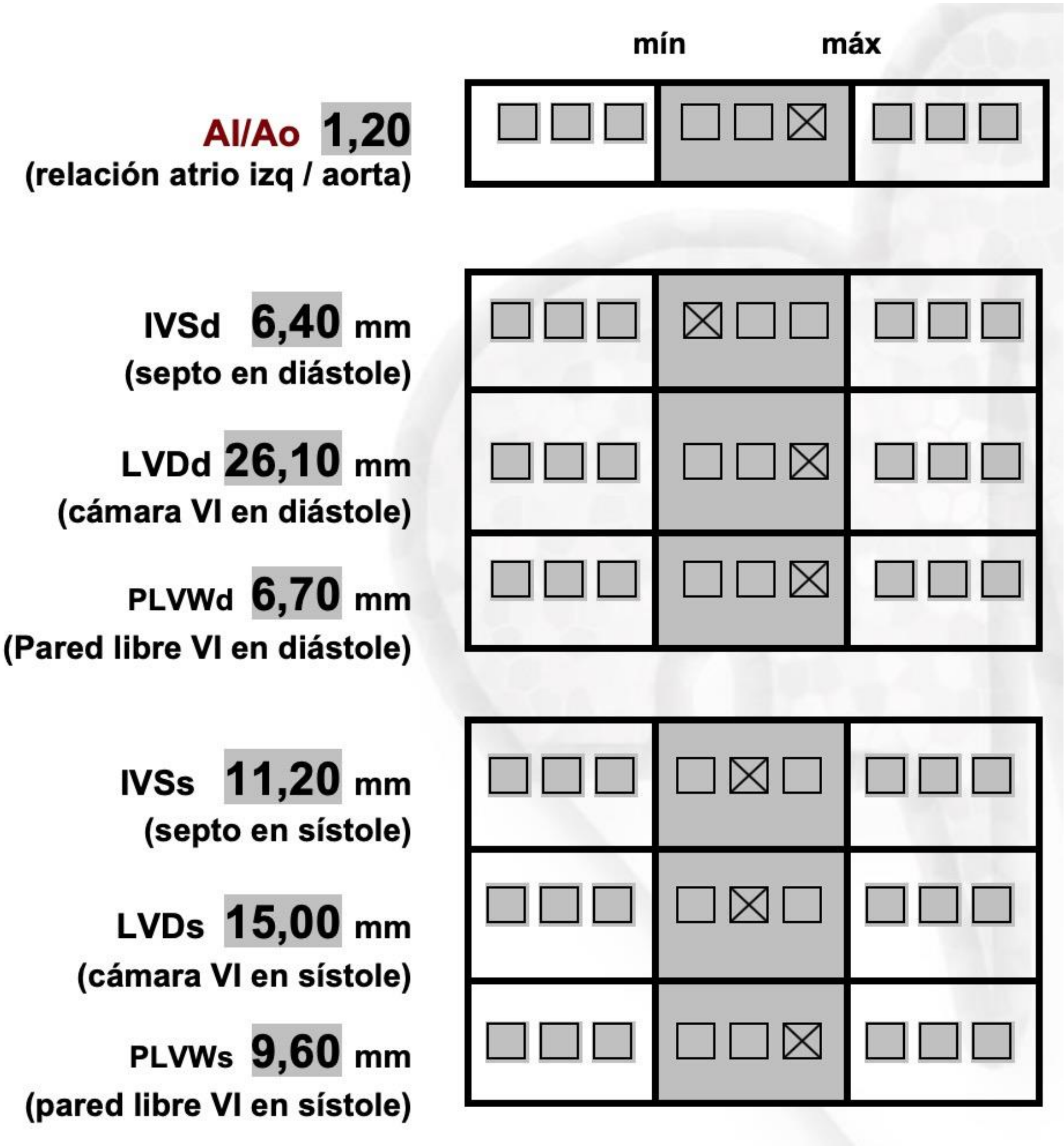

2a- **How would you describe the size of the LEFT ATRIUM?***

- Normal
- Increased
- Decreased

2b- **How would you describe the size of the INTERVENTRICULAR SEPTUM in DIASTOLE?***

- Normal
- Increased
- Decreased

2c- **How would you describe the size of the LEFT VENTRICULAR CHAMBER in DIASTOLE?***

- Normal
- Increased
- Decreased

2d- **How would you describe the size of the LEFT VENTRICULAR FREE WALL in DIASTOLE?***

- Normal

- Increased
- Decreased

2e- **How would you describe the size of the INTERVENTRICULAR SEPTUM in SYSTOLE?***

- Normal
- Increased
- Decreased

2f- **How would you describe the size of the LEFT VENTRICULAR CHAMBER in SYSTOLE?***

- Normal
- Increased
- Decreased

2g- **How would you describe the size of the LEFT VENTRICULAR FREE WALL in SYSTOLE?***

- Normal
- Increased
- Decreased

Third Clinical Case

Interpret the following clinical case of a 12 kg mixed-breed dog.

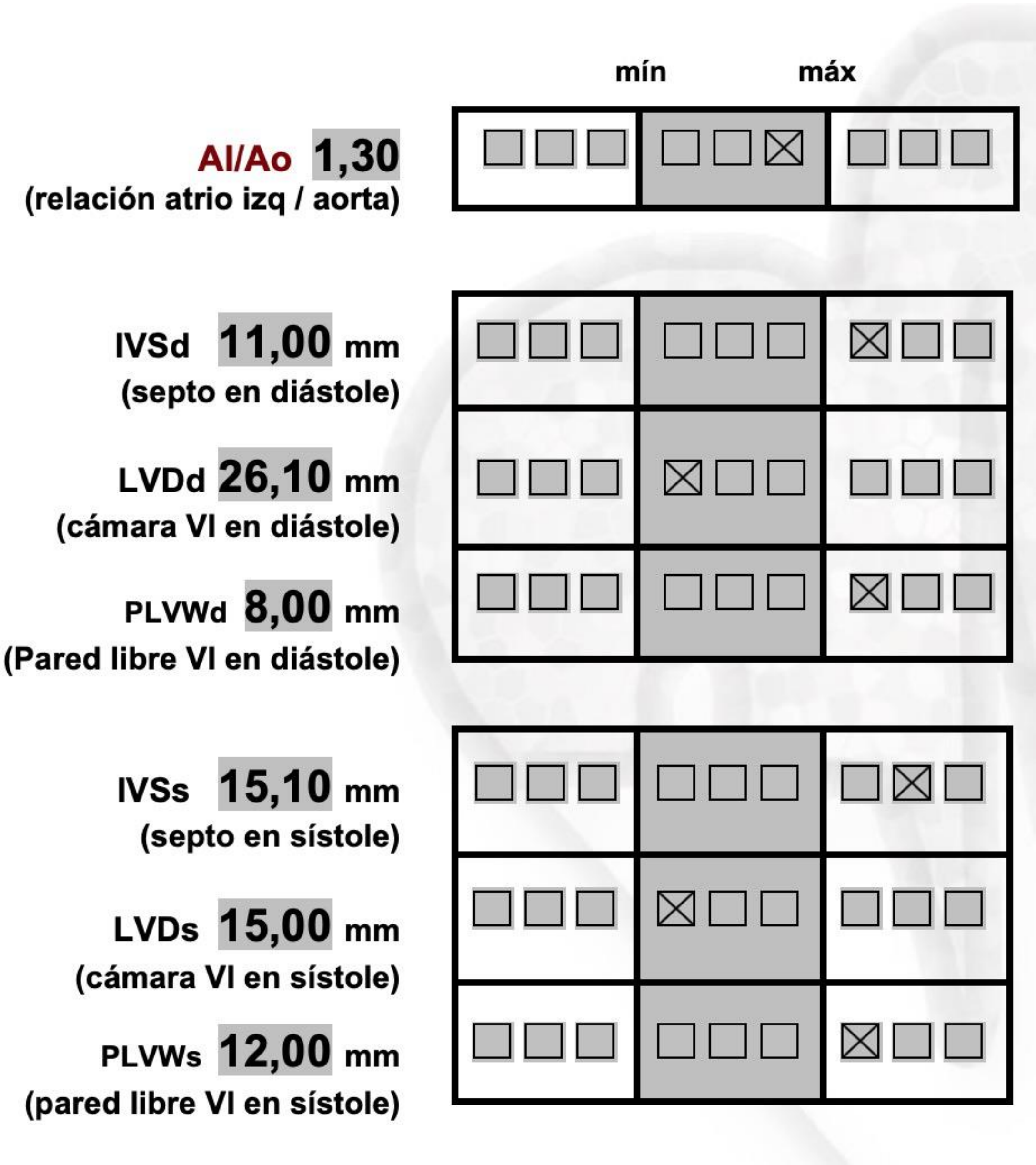

3a- **How would you describe the size of the LEFT ATRIUM?***

- Normal
- Increased
- Decreased

3b- **How would you describe the size of the INTERVENTRICULAR SEPTUM in DIASTOLE?***

- Normal
- Increased
- Decreased

3c- **How would you describe the size of the LEFT VENTRICULAR CHAMBER in DIASTOLE?***

- Normal
- Increased

- Decreased

3d- **How would you describe the size of the LEFT VENTRICULAR FREE WALL in DIASTOLE?***

- Normal
- Increased
- Decreased

3e- **How would you describe the size of the INTERVENTRICULAR SEPTUM in SYSTOLE?***

- Normal
- Increased
- Decreased

3f- **How would you describe the size of the LEFT VENTRICULAR CHAMBER in SYSTOLE?***

- Normal
- Increased
- Decreased

3g- **How would you describe the size of the LEFT VENTRICULAR FREE WALL in SYSTOLE?***

- Normal
- Increased
- Decreased

Final Clinical Case

Interpret the following clinical case of a 7 kg Yorkshire Terrier.

Medición	Caso "alma" yorkshire 7 kg	2.5-5 kg	5-10 kg	10-14 kg	14-17 kg	17-18 kg
**AI / Ao** (atrio aorta)	2,2	1 – 1,4	1 – 1,4	1 – 1,4	1 – 1,4	1 – 1,4
**IVSd** (septo en diástole)	6,1	4,8 -7,7	6,0 - 8,4	7,1 - 8,9	7,8 - 9,3	8,3 - 9,4
**LVDd** (cámara VI en diástole)	33,1	11,8 -25	20,2 - 28,9	25,3 - 31,7	28,7 -33,8	31,1 -34,4
**PLVWd** (pared libre VI en diástole)	7,2	3,75 - 6,8	4,8 - 6,8	5,7 - 7,2	6,3 - 7,5	6,7 - 7,6
**IVSs** (septo en sístole)	10,2	7,1 - 12,1	9,0 - 12,1	10,7 - 13,0	11,8 -13,6	12,5 -13,8
**LVDs** (cámara VI en sístole)	19,2	5,3 - 14,3	11,2 - 17,3	14,7 - 19,2	17,0 -20,6	18,7 -21,0
**PLVWs** (pared libre VI en sístole)	11,6	6,1 - 9,8	8,0 - 10,7	9,4 - 11,4	10,3 -11,9	10,9 -12,0

4a- **How would you describe the size of the LEFT ATRIUM?***

- Normal
- Increased
- Decreased

4b- **How would you describe the size of the INTERVENTRICULAR SEPTUM in DIASTOLE?***

- Normal
- Increased
- Decreased

4c- **How would you describe the size of the LEFT VENTRICULAR CHAMBER in DIASTOLE?***

- Normal
- Increased
- Decreased

4d- **How would you describe the size of the LEFT VENTRICULAR FREE WALL in DIASTOLE?***

- Normal
- Increased
- Decreased

4e- **How would you describe the size of the INTERVENTRICULAR SEPTUM in SYSTOLE?***

- Normal
- Increased

- Decreased

4f- **How would you describe the size of the LEFT VENTRICULAR CHAMBER in SYSTOLE?***

- Normal
- Increased
- Decreased

4g- **How would you describe the size of the LEFT VENTRICULAR FREE WALL in SYSTOLE?***

- Normal
- Increased
- Decreased

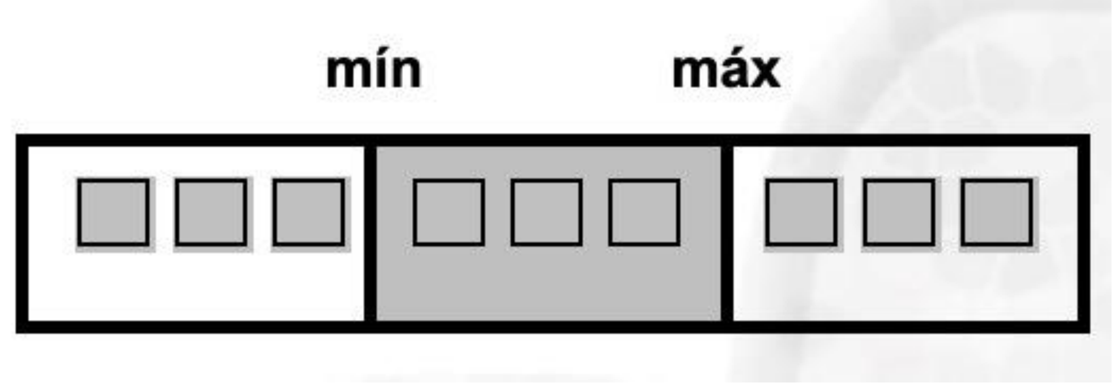

- **In your opinion, what is the EASE OF USE of the graphical representation tool we worked with?**
  (You can choose any range, knowing that 0 is difficult to use, and 5 is very easy to interpret.)

- **In your opinion, what is the LEVEL OF EFFECTIVENESS of the graphical representation tool we worked with?**
  (You can choose any range, knowing that 0 seems ineffective for describing changes, and 5 seems effective for describing changes.)

- **Would you like to see the graphical box system implemented in future studies?***
  - Yes, it would provide valuable information for my patients.
  - Maybe, I am not yet convinced of its usefulness.
  - No, it is irrelevant for most cases.

Formulario CARDIOBOX en español (ORIGINAL)

Interpretación ecocardiográfica básica

(copia de https://form.jotform.com/223278319852059)

Actividad para especialistas y clínicos veterinarios.

Introducción a la actividad

Por favor te pediremos que veas este breve video donde te explicaremos mínimamente los pasos básicos que vamos a pedirte.

https://youtu.be/U5pup4iB840

Formulario anónimo - interpretación básica de reportes ecocardiográficos

No te pediremos tu nombre, solo tendrás que completar los datos de este formulario con mínima información privada de interés demográfico. Demorarás muy pocos minutos en completar este ejercicio.

¿Cuántos años tienes?*

¿Dónde vives?*

Ciudad

País

¿Cuál es tu formación académica?*

Estudiante de veterinaria

Veterinario/a clínico/a

Cardiólogo/a veterinario/a

Anestesiólogo/a veterinario/a

Otra especialidad veterinaria

No tengo formación veterinaria

Primer caso clínico

Deberá interpretar el siguiente caso clínico de un caniche de 5,5 kg. Tiene los valores de parámetros ecocardiográficos básicos en la segunda columna y deberá compararlos con los valores de referencia de su peso.

Medición	Caso "troy" caniche 5,5 kg	2.5-5 kg	5-10 kg	10-14 kg	14-17 kg	17-18 kg
**AI / Ao** (atrio aorta)	1,2	1 – 1,4	1 – 1,4	1 – 1,4	1 – 1,4	1 – 1,4
**IVSd** (septo en diástole)	6,4	4,8 -7,7	6,0 - 8,4	7,1 - 8,9	7,8 - 9,3	8,3 - 9,4
**LVDd** (cámara VI en diástole)	26,1	11,8 -25	20,2 - 28,9	25,3 - 31,7	28,7 -33,8	31,1 -34,4
**PLVWd** (pared libre VI en diástole)	6,7	3,75 - 6,8	4,8 - 6,8	5,7 - 7,2	6,3 - 7,5	6,7 - 7,6
**IVSs** (septo en sístole)	11,2	7,1 - 12,1	9,0 - 12,1	10,7 - 13,0	11,8 -13,6	12,5 -13,8
**LVDs** (cámara VI en sístole)	15,0	5,3 - 14,3	11,2 - 17,3	14,7 - 19,2	17,0 -20,6	18,7 -21,0
**PLVWs** (pared libre VI en sístole)	9,6	6,1 - 9,8	8,0 - 10,7	9,4 - 11,4	10,3 -11,9	10,9 -12,0

1a- ¿Cómo se encuentra el tamaño del ATRIO IZQUIERDO?*

Normal

Aumentado

Disminuido

1b- ¿Cómo se encuentra el tamaño del SEPTO INTERVENTRICULAR en DIASTOLE?*

Normal

Aumentado

Disminuido

1c- ¿Cómo se encuentra el tamaño de la CÁMARA VENTRICULAR IZQUIERDA en DIASTOLE?*

Normal

Aumentado

Disminuido

1d- ¿Cómo se encuentra el tamaño de la PARED LIBRE ventricular izquierda en DIASTOLE?*

Normal

Aumentado

Disminuido

1e- ¿Cómo se encuentra el tamaño del SEPTO INTERVENTRICULAR en SISTOLE?*

Normal

Aumentado

Disminuido

1f- ¿Cómo se encuentra el tamaño de la CÁMARA VENTRICULAR IZQUIERDA en SISTOLE?*

Normal

Aumentado

Disminuido

1g- ¿Cómo se encuentra el tamaño de la PARED LIBRE ventricular izquierda en SISTOLE?

Normal

Aumentado

Disminuido

Segundo Caso clínico

Deberá interpretar el siguiente caso clínico de un maltes de 7 kg. Los rangos de referencia se dan en los cuadros gráficos.

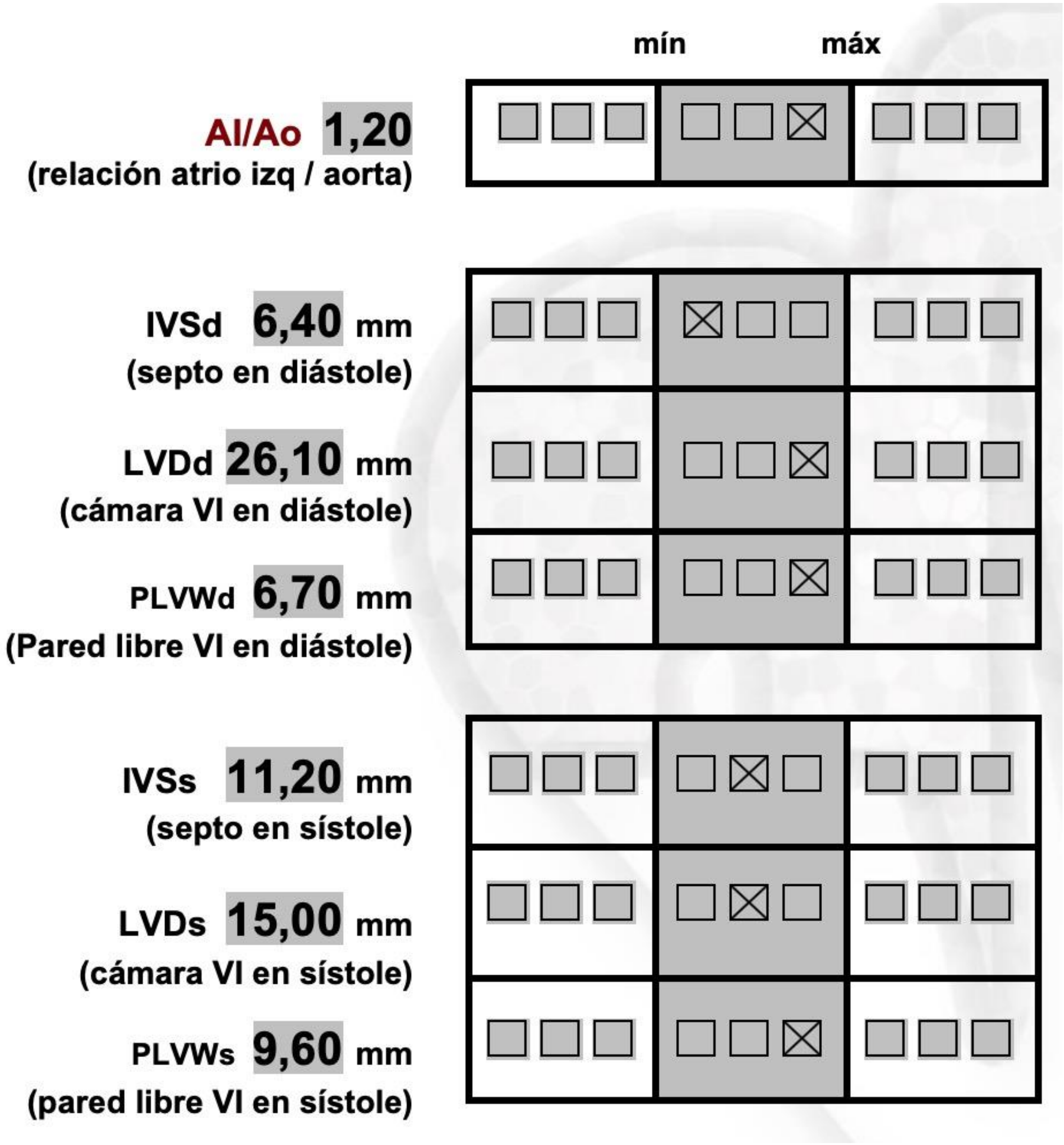

2a- ¿Cómo se encuentra el tamaño del ATRIO IZQUIERDO?*

Normal

Aumentado

Disminuido

2b- ¿Cómo se encuentra el tamaño del SEPTO INTERVENTRICULAR en DIASTOLE?*

Normal

Aumentado

Disminuido

2c- ¿Cómo se encuentra el tamaño de la CÁMARA VENTRICULAR IZQUIERDA en DIASTOLE?*

Normal

Aumentado

Disminuido

2d- ¿Cómo se encuentra el tamaño de la PARED LIBRE ventricular izquierda en DIASTOLE?*

Normal

Aumentado

Disminuido

2e- ¿Cómo se encuentra el tamaño del SEPTO INTERVENTRICULAR en SISTOLE?*

Normal

Aumentado

Disminuido

2f- ¿Cómo se encuentra el tamaño de la CÁMARA VENTRICULAR IZQUIERDA en SISTOLE?*

Normal

Aumentado

Disminuido

2g- ¿Cómo se encuentra el tamaño de la PARED LIBRE ventricular izquierda en SISTOLE?*

Normal

Aumentado

Disminuido

Tercer caso clínico

Deberá interpretar el siguiente caso clínico de un canino mestizo de 12 kg.

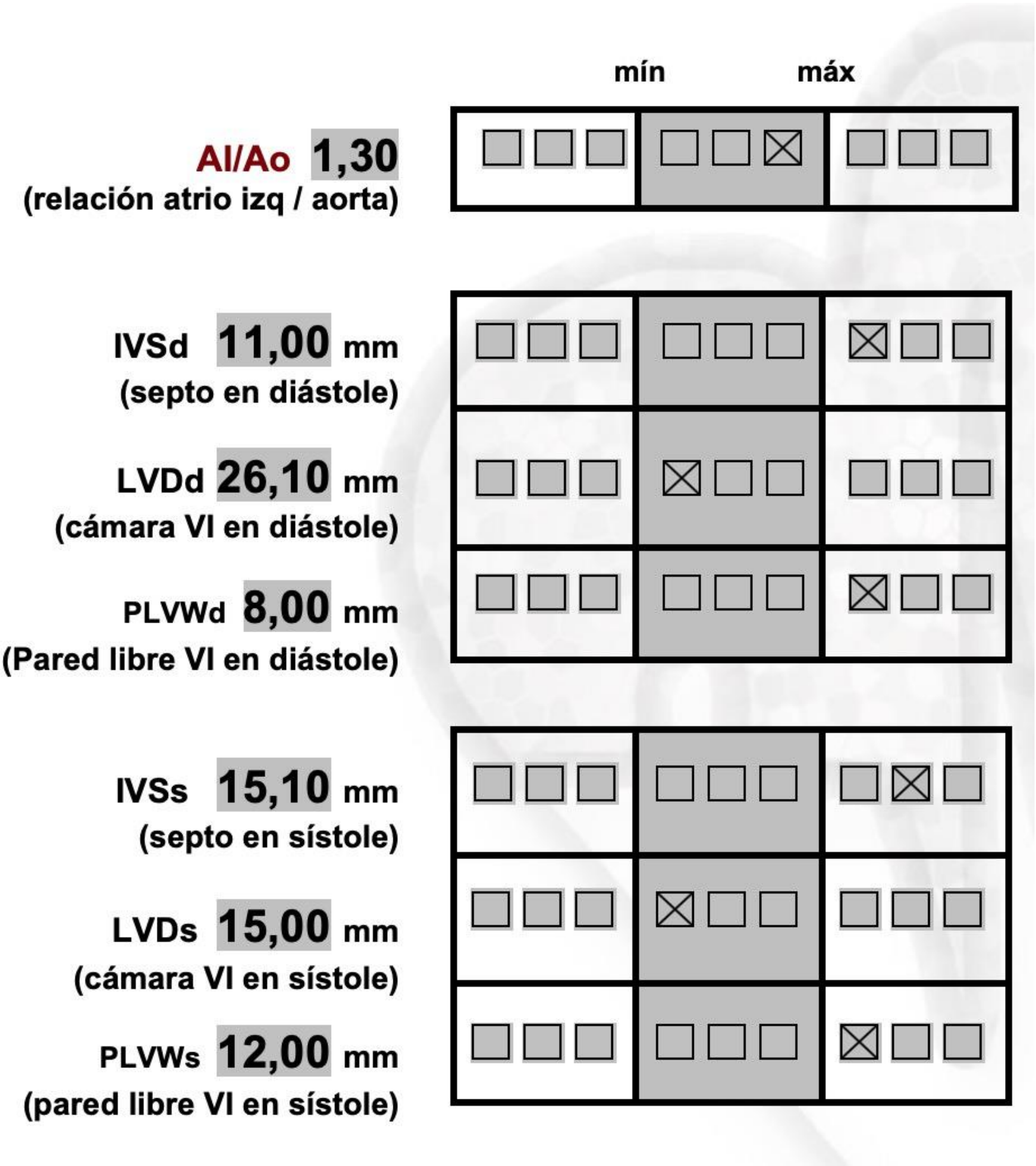

3a- ¿Cómo se encuentra el tamaño del ATRIO IZQUIERDO?*

NormalAumentadoDisminuido

3b- ¿Cómo se encuentra el tamaño del SEPTO INTERVENTRICULAR en DIASTOLE?*

Normal

Aumentado

Disminuido

3c- ¿Cómo se encuentra el tamaño de la CÁMARA VENTRICULAR IZQUIERDA en DIASTOLE?*

Normal

Aumentado

Disminuido

3d- ¿Cómo se encuentra el tamaño de la PARED LIBRE ventricular izquierda en DIASTOLE?*

Normal

Aumentado

Disminuido

3e- ¿Cómo se encuentra el tamaño del SEPTO INTERVENTRICULAR en SISTOLE?*

Normal

Aumentado

Disminuido

3f- ¿Cómo se encuentra el tamaño de la CÁMARA VENTRICULAR IZQUIERDA en SISTOLE?*

Normal

Aumentado

Disminuido

3g- ¿Cómo se encuentra el tamaño de la PARED LIBRE ventricular izquierda en SISTOLE?*

Normal

Aumentado

Disminuido

Último caso clínico

Deberá interpretar el siguiente caso clínico de un yorkshire de 7 kg.

Medición	Caso "alma" yorkshire 7 kg	2.5-5 kg	5-10 kg	10-14 kg	14-17 kg	17-18 kg
AI / Ao (atrio aorta)	2,2	1 – 1,4	1 – 1,4	1 – 1,4	1 – 1,4	1 – 1,4
IVSd (septo en diástole)	6,1	4,8 -7,7	6,0 - 8,4	7,1 - 8,9	7,8 - 9,3	8,3 - 9,4
LVDd (cámara VI en diástole)	33,1	11,8 -25	20,2 - 28,9	25,3 - 31,7	28,7 -33,8	31,1 -34,4
PLVWd (pared libre VI en diástole)	7,2	3,75 - 6,8	4,8 - 6,8	5,7 - 7,2	6,3 - 7,5	6,7 - 7,6
IVSs (septo en sístole)	10,2	7,1 - 12,1	9,0 - 12,1	10,7 - 13,0	11,8 -13,6	12,5 -13,8
LVDs (cámara VI en sístole)	19,2	5,3 - 14,3	11,2 - 17,3	14,7 - 19,2	17,0 -20,6	18,7 -21,0
PLVWs (pared libre VI en sístole)	11,6	6,1 - 9,8	8,0 - 10,7	9,4 - 11,4	10,3 -11,9	10,9 -12,0

4a- ¿Cómo se encuentra el tamaño del ATRIO IZQUIERDO?*

Normal

Aumentado

Disminuido

4b- ¿Cómo se encuentra el tamaño del SEPTO INTERVENTRICULAR en DIASTOLE?*

Normal

Aumentado

Disminuido

4c- ¿Cómo se encuentra el tamaño de la CÁMARA VENTRICULAR IZQUIERDA en DIASTOLE?*

Normal

Aumentado

Disminuido

4d- ¿Cómo se encuentra el tamaño de la PARED LIBRE ventricular izquierda en DIASTOLE?*

NormalAumentadoDisminuido

4e- ¿Cómo se encuentra el tamaño del SEPTO INTERVENTRICULAR en SISTOLE?*

Normal

Aumentado

Disminuido

4f- ¿Cómo se encuentra el tamaño de la CÁMARA VENTRICULAR IZQUIERDA en SISTOLE?*

Norma

lAumentado

Disminuido

4g- ¿Cómo se encuentra el tamaño de la PARED LIBRE ventricular izquierda en SISTOLE?*

Normal

Aumentado

Disminuido

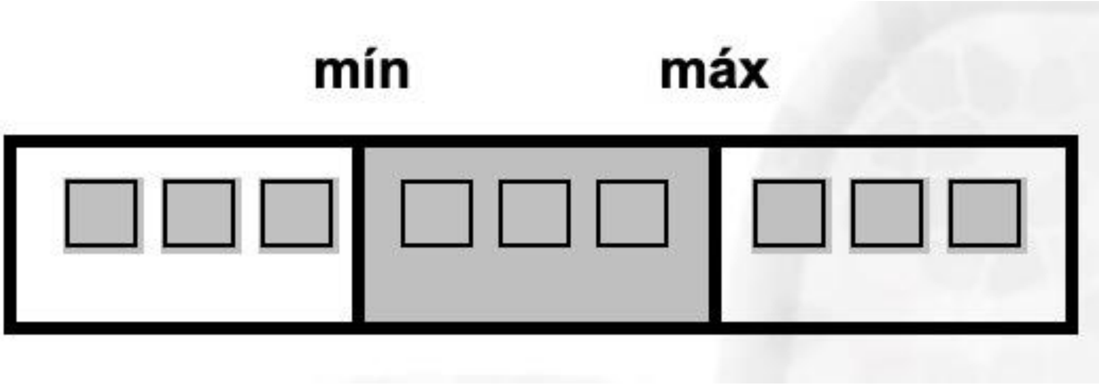

¿Cuál es a tu criterio el GRADO DE FACILIDAD DE USO de la herramienta del cuadro de representación gráfica que hemos trabajado? (puedes elegir cualquier rango sabiendo que: 0 es difícil de usar; 5 es muy fácil interpretación)*

¿Cuál es a tu criterio el NIVEL DE EFICACIA de la herramienta del cuadro de representación gráfica que hemos trabajado? puedes elegir cualquier rango sabiendo que: 0 parece poco eficaz para describir modificaciones; 5 parece eficaz para describir modificaciones)*

¿Te gustaría ver implementado el sistema gráfico de cajas futuros estudios?*

Si, me aportaría información en mis pacientes

Tal vez, todavía no estoy convencido de su utilidad.

No, es irrelevante para la mayoría de los casos
