# Supplementary material for: A New Graphical Method for Displaying Two-Dimensional Echocardiography Results in Dogs: Comprehensive Analysis of Results of Diagnostic Imaging Organized in a BOX (CARDIOBOX)
Source: Vet Sci. 2025 Jan 9;12(1):34. doi: 10.3390/vetsci12010034 (PMC11769013; doi:10.3390/vetsci12010034)
Supplement: Supplementary file 1 [file vetsci-12-00034-s001.zip › Figure S4-CARDIOBOX REPORT.pdf]

CardioBox Report

Fecha: 02/12/2024 11:49:00

OWNER(S)

Name and Last Name	NAME LAST NAME
E-mail	

PATIENT

HC	Name
Sp. .	Breed

AGE (years)	SEX	Weight (KG)	BSA (m^2)	BODY CONDITION SCORTE	SIZE
	.			.	.

CHIEF COMPLAINT:

RECENT HISTORY ☐ FATIGUE ☐ COUGH ☐ ASCITES ☐ WEAKNESS ☐ SYNCOPE

GENERAL INSPECTION:

Mucous Membrane color: Pink **CRT:** " **HYDRATATION:** Normal

PULSE: **OTHER CLINICAL SIGNS:**

RESPIRATORY PATTERN: Normal

COUGH REFLEX: Normal

AUSCULTATION

Cardiac Area:

Pulmonary Area:

PRESIÓN ARTERIAL (mmHg)

Systolic BP: **Mean BP:** **Diastolic BP:** **HR:**

Electrocardiogram (EKG) Findings:

Echocardiogram Report (TTE)

Atria:

Right Ventricle:

Left Ventricel:

Mediastinum, Pleural space and Lungs:

		CARDIOBOX ® mín		máx
AORTA	mm			
Left Atria	mm			
LA/Ao		☐ ☐ ☐	☐ ☐ ☐	☐ ☐ ☐
VDd	mm			
IVSd	mm	☐ ☐ ☐	☐ ☐ ☐	☐ ☐ ☐
LVIDd	mm	☐ ☐ ☐	☐ ☐ ☐	☐ ☐ ☐
PLVWDd	mm	☐ ☐ ☐	☐ ☐ ☐	☐ ☐ ☐
IVSs	mm	☐ ☐ ☐	☐ ☐ ☐	☐ ☐ ☐
LVIDs	mm	☐ ☐ ☐	☐ ☐ ☐	☐ ☐ ☐
PLVWs	mm	☐ ☐ ☐	☐ ☐ ☐	☐ ☐ ☐

ÍNDICES

EPSS mm

AF %

EF %

RWT:

LV Mass 0,00 g
(Troy´s Fórmula)

LV MASS / kg:
(g/kg)

Mass Index:
(g/m^2)

RV/LV

DIAGNOSIS (PRESUMPTIVE/DEFINITIVE): .

Recommendations:

Dr:
