# Supplementary material for: A New Graphical Method for Displaying Two-Dimensional Echocardiography Results in Dogs: Comprehensive Analysis of Results of Diagnostic Imaging Organized in a BOX (CARDIOBOX)
Source: Vet Sci. 2025 Jan 9;12(1):34. doi: 10.3390/vetsci12010034 (PMC11769013; doi:10.3390/vetsci12010034)
Supplement: Supplementary file 1 [file vetsci-12-00034-s001.zip › Table S2-cardiobox table 2-17kg.pdf]

CARDIOBOX TABLES WITH PREDICTION INTERVALS FEATURING WEIGHT-BASED REFERENCE VALUES FOR CANINE ECHOCARDIOGRAPI

WEIGHT (kg)
2,00

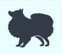

parameter	A	B	C	D	E	F	G	H	I	J	K	L	M	N	O	P	Q	R
							min					max						
Aid	4,39	5,24	5,36	6,08	6,21	6,93	7,05	8,99	9,12	10,92	11,05	12,86	12,99	16,01	16,14	19,30	19,42	28,22
IVSd	2,62	2,83	2,95	3,04	3,16	3,25	3,37	4,52	4,64	5,68	5,80	6,83	6,95	7,20	7,32	7,68	7,80	9,92
LVDd	8,35	10,06	10,19	11,78	11,90	13,49	13,61	16,01	16,13	18,40	18,52	20,79	20,91	25,41	25,54	30,16	30,28	37,21
PLVWd	2,18	2,44	2,56	2,70	2,82	2,96	3,08	4,34	4,46	5,60	5,71	6,86	6,97	7,19	7,30	7,63	7,75	10,04
IVSs	1,35	2,53	2,65	3,71	3,83	4,89	5,01	6,55	6,67	8,10	8,22	9,65	9,76	10,08	10,20	10,63	10,75	14,39
LVDs	2,98	3,80	3,92	4,61	4,74	5,43	5,55	8,06	8,18	10,57	10,69	13,07	13,20	16,30	16,43	19,66	19,78	31,75
PLVWs	3,77	4,08	4,20	4,39	4,50	4,70	4,81	6,37	6,49	7,93	8,04	9,48	9,60	9,87	9,99	10,38	10,50	13,06

CARDIOBOX Table for Canine Patients Weighing 2 kg. Use it exclusively with proper citation of the source in your echocardiographic report. © 2024

Source: Curra-Gagliano FJ, Engel-Manchado J, Ceballos M, Redondo I A new graphical method for displaying two-dimensional echocardiography results in dogs: Comprehensive Analysis of Results of Diagnostic Imaging Organized in a BOX (CARDIOBOX). 2024.

WEIGHT (kg)
3,00

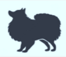

parameter	A	B	C	D	E	F	G	H	I	J	K	L	M	N	O	P	Q	R
							min					max						
Aid	5,05	6,02	6,17	6,99	7,14	7,97	8,11	10,34	10,48	12,56	12,71	14,79	14,93	18,42	18,56	22,19	22,34	32,45
IVSd	2,89	3,12	3,25	3,36	3,49	3,59	3,72	4,99	5,12	6,26	6,39	7,53	7,66	7,94	8,07	8,47	8,60	10,94
LVDd	9,41	11,34	11,48	13,27	13,41	15,20	15,34	18,03	18,17	20,73	20,86	23,42	23,56	28,63	28,77	33,98	34,12	41,93
PLVWd	2,40	2,68	2,81	2,97	3,10	3,25	3,38	4,77	4,89	6,15	6,28	7,53	7,66	7,89	8,02	8,39	8,52	11,03
IVSs	1,48	2,79	2,92	4,09	4,22	5,39	5,52	7,22	7,35	8,93	9,06	10,63	10,76	11,11	11,24	11,72	11,85	15,86
LVDs	3,38	4,31	4,45	5,24	5,38	6,17	6,31	9,16	9,30	12,01	12,15	14,85	15,00	18,52	18,66	22,33	22,47	36,07
PLVWs	4,13	4,46	4,59	4,80	4,93	5,14	5,27	6,97	7,10	8,67	8,80	10,38	10,50	10,80	10,93	11,36	11,49	14,28

CARDIOBOX Table for Canine Patients Weighing 3 kg. Use it exclusively with proper citation of the source in your echocardiographic report. © 2024

Source: Curra-Gagliano FJ, Engel-Manchado J, Ceballos M, Redondo I A new graphical method for displaying two-dimensional echocardiography results in dogs: Comprehensive Analysis of Results of Diagnostic Imaging Organized in a BOX (CARDIOBOX). 2024.

WEIGHT (kg)
5,00

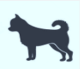

parameter	A	B	C	D	E	F	G	H	I	J	K	L	M	N	O	P	Q	R
							min					max						
Aid	6,02	7,18	7,36	8,34	8,52	9,50	9,68	12,33	12,51	14,98	15,16	17,64	17,81	21,97	22,14	26,47	26,64	38,71
IVSd	3,27	3,53	3,68	3,80	3,94	4,06	4,20	5,64	5,79	7,08	7,23	8,52	8,67	8,98	9,12	9,58	9,73	12,38
LVDd	10,93	13,17	13,34	15,42	15,58	17,66	17,82	20,95	21,12	24,09	24,25	27,22	27,38	33,27	33,43	39,49	39,65	48,72
PLVWd	2,70	3,02	3,17	3,34	3,49	3,66	3,81	5,37	5,51	6,92	7,07	8,48	8,62	8,89	9,03	9,44	9,59	12,42
IVSs	1,68	3,15	3,30	4,62	4,77	6,09	6,24	8,17	8,31	10,09	10,24	12,02	12,16	12,56	12,70	13,24	13,39	17,92
LVDs	3,98	5,07	5,23	6,15	6,32	7,24	7,41	10,76	10,92	14,10	14,27	17,45	17,61	21,76	21,92	26,23	26,40	42,37
PLVWs	4,62	5,00	5,14	5,38	5,52	5,75	5,90	7,81	7,95	9,71	9,86	11,62	11,77	12,10	12,24	12,72	12,87	16,00

CARDIOBOX Table for Canine Patients Weighing 5 kg. Use it exclusively with proper citation of the source in your echocardiographic report. © 2024

Source: Curra-Gagliano FJ, Engel-Manchado J, Ceballos M, Redondo I A new graphical method for displaying two-dimensional echocardiography results in dogs: Comprehensive Analysis of Results of Diagnostic Imaging Organized in a BOX (CARDIOBOX). 2024.

WEIGHT (kg)
7,50

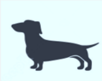

parameter	A	B	C	D	E	F	G	H	I	J	K	L	M	N	O	P	Q	R
							min					max						
Aid	6,93	8,26	8,46	9,60	9,80	10,93	11,13	14,18	14,38	17,23	17,44	20,29	20,49	25,27	25,47	30,44	30,64	44,52
IVSd	3,61	3,90	4,06	4,18	4,35	4,47	4,64	6,22	6,38	7,81	7,97	9,40	9,56	9,90	10,06	10,56	10,73	13,65
LVDd	12,31	14,84	15,02	17,37	17,55	19,90	20,08	23,61	23,79	27,13	27,32	30,66	30,84	37,48	37,66	44,48	44,66	54,89
PLVWd	2,97	3,32	3,48	3,67	3,83	4,02	4,18	5,89	6,05	7,60	7,76	9,32	9,47	9,76	9,92	10,37	10,53	13,65
IVSs	1,85	3,47	3,63	5,09	5,26	6,71	6,88	9,00	9,16	11,12	11,28	13,25	13,41	13,84	14,00	14,60	14,76	19,76
LVDs	4,52	5,76	5,94	6,99	7,18	8,23	8,42	12,22	12,41	16,02	16,21	19,82	20,01	24,72	24,91	29,81	29,99	48,14
PLVWs	5,06	5,47	5,63	5,88	6,04	6,30	6,45	8,54	8,70	10,63	10,79	12,72	12,87	13,24	13,40	13,92	14,08	17,51

CARDIOBOX Table for Canine Patients Weighing 7,5 kg. Use it exclusively with proper citation of the source in your echocardiographic report. © 2024

Source: Curra-Gagliano FJ, Engel-Manchado J, Ceballos M, Redondo I A new graphical method for displaying two-dimensional echocardiography results in dogs: Comprehensive Analysis of Results of Diagnostic Imaging Organized in a BOX (CARDIOBOX). 2024.

WEIGHT (kg)
10,00

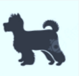

parameter	A	B	C	D	E	F	G	H	I	J	K	L	M	N	O	P	Q	R
							min					max						
Aid	7,65	9,12	9,34	10,60	10,82	12,07	12,29	15,66	15,88	19,03	19,25	22,40	22,63	27,90	28,12	33,62	33,84	49,16
IVSd	3,87	4,18	4,35	4,49	4,66	4,79	4,97	6,67	6,84	8,37	8,54	10,07	10,24	10,61	10,78	11,32	11,50	14,63
LVDd	13,40	16,15	16,35	18,90	19,10	21,66	21,85	25,69	25,89	29,53	29,73	33,37	33,56	40,79	40,99	48,41	48,61	59,73
PLVWd	3,17	3,55	3,72	3,93	4,10	4,30	4,47	6,30	6,47	8,13	8,30	9,96	10,13	10,44	10,61	11,09	11,26	14,59
IVSs	1,98	3,72	3,89	5,46	5,63	7,19	7,37	9,64	9,82	11,92	12,09	14,19	14,37	14,83	15,00	15,64	15,81	21,17
LVDs	4,95	6,30	6,51	7,66	7,86	9,01	9,22	13,38	13,59	17,54	17,75	21,70	21,91	27,07	27,27	32,63	32,84	52,71
PLVWs	5,39	5,83	6,00	6,27	6,44	6,71	6,88	9,10	9,27	11,33	11,50	13,56	13,72	14,11	14,28	14,84	15,01	18,66

CARDIOBOX Table for Canine Patients Weighing 10 kg. Use it exclusively with proper citation of the source in your echocardiographic report. © 2024

Source: Curra-Gagliano FJ, Engel-Manchado J, Ceballos M, Redondo I A new graphical method for displaying two-dimensional echocardiography results in dogs: Comprehensive Analysis of Results of Diagnostic Imaging Organized in a BOX (CARDIOBOX). 2024.

WEIGHT (kg)
12,50

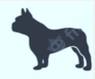

parameter	A	B	C	D	E	F	G	H	I	J	K	L	M	N	O	P	Q	R
							min					max						
Aid	8,26	9,85	10,09	11,44	11,68	13,04	13,27	16,92	17,15	20,56	20,80	24,20	24,44	30,13	30,37	36,31	36,55	53,10
IVSd	4,08	4,41	4,59	4,73	4,92	5,06	5,24	7,04	7,22	8,83	9,02	10,63	10,81	11,19	11,38	11,95	12,13	15,44
LVDd	14,31	17,25	17,46	20,19	20,40	23,12	23,33	27,43	27,64	31,53	31,74	35,63	35,84	43,56	43,77	51,69	51,90	63,78
PLVWd	3,34	3,74	3,92	4,13	4,31	4,53	4,71	6,64	6,82	8,56	8,74	10,49	10,67	10,99	11,17	11,68	11,86	15,37
IVSs	2,09	3,92	4,11	5,76	5,94	7,59	7,77	10,17	10,36	12,57	12,76	14,97	15,16	15,65	15,83	16,50	16,68	22,33
LVDs	5,31	6,76	6,98	8,21	8,44	9,67	9,89	14,36	14,58	18,82	19,04	23,29	23,51	29,04	29,26	35,01	35,23	56,54
PLVWs	5,66	6,13	6,30	6,59	6,77	7,05	7,23	9,57	9,74	11,91	12,08	14,24	14,42	14,83	15,01	15,59	15,77	19,61

CARDIOBOX Table for Canine Patients Weighing 12,5 kg. Use it exclusively with proper citation of the source in your echocardiographic report. © 2024

Source: Curra-Gagliano FJ, Engel-Manchado J, Ceballos M, Redondo I A new graphical method for displaying two-dimensional echocardiography results in dogs: Comprehensive Analysis of Results of Diagnostic Imaging Organized in a BOX (CARDIOBOX). 2024.

WEIGHT (kg)
15,00

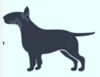

parameter	A	B	C	D	E	F	G	H	I	J	K	L	M	N	O	P	Q	R
							min					max						
Aid	8,80	10,49	10,75	12,19	12,44	13,88	14,14	18,01	18,27	21,89	22,15	25,77	26,02	32,09	32,35	38,67	38,92	56,54
IVSd	4,26	4,61	4,80	4,95	5,14	5,29	5,48	7,35	7,55	9,23	9,42	11,10	11,30	11,70	11,89	12,48	12,68	16,13
LVDd	15,10	18,20	18,42	21,30	21,52	24,40	24,62	28,94	29,17	33,27	33,49	37,59	37,81	45,95	46,18	54,54	54,76	67,30
PLVWd	3,48	3,90	4,09	4,31	4,50	4,73	4,91	6,92	7,11	8,93	9,12	10,94	11,13	11,47	11,66	12,18	12,37	16,03
IVSs	2,18	4,10	4,29	6,01	6,21	7,93	8,12	10,63	10,82	13,14	13,33	15,64	15,83	16,35	16,54	17,24	17,43	23,33
LVDs	5,62	7,16	7,39	8,70	8,93	10,24	10,47	15,20	15,44	19,93	20,17	24,66	24,90	30,75	30,99	37,08	37,31	59,89
PLVWs	5,90	6,38	6,56	6,86	7,04	7,34	7,53	9,96	10,14	12,40	12,58	14,83	15,02	15,44	15,63	16,24	16,42	20,42

CARDIOBOX Table for Canine Patients Weighing 15 kg. Use it exclusively with proper citation of the source in your echocardiographic report. © 2024

Source: Curra-Gagliano FJ, Engel-Manchado J, Ceballos M, Redondo I A new graphical method for displaying two-dimensional echocardiography results in dogs: Comprehensive Analysis of Results of Diagnostic Imaging Organized in a BOX (CARDIOBOX). 2024.

WEIGHT (kg)
17,50

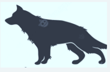

parameter	A	B	C	D	E	F	G	H	I	J	K	L	M	N	O	P	Q	R
							min					max						
Aid	9,28	11,07	11,33	12,85	13,12	14,64	14,91	19,00	19,27	23,09	23,35	27,18	27,44	33,84	34,11	40,78	41,05	59,63
IVSd	4,43	4,78	4,98	5,13	5,33	5,49	5,69	7,63	7,83	9,58	9,78	11,52	11,72	12,14	12,34	12,96	13,16	16,74
LVDd	15,80	19,04	19,27	22,29	22,52	25,53	25,76	30,29	30,52	34,81	35,04	39,34	39,57	48,09	48,32	57,07	57,30	70,42
PLVWd	3,61	4,04	4,23	4,47	4,66	4,90	5,09	7,18	7,37	9,26	9,45	11,34	11,53	11,89	12,08	12,63	12,82	16,61
IVSs	2,27	4,25	4,45	6,24	6,44	8,23	8,43	11,03	11,23	13,63	13,83	16,23	16,43	16,96	17,16	17,89	18,09	24,21
LVDs	5,90	7,52	7,76	9,13	9,38	10,75	11,00	15,96	16,21	20,92	21,17	25,89	26,14	32,28	32,53	38,92	39,17	62,87
PLVWs	6,10	6,60	6,79	7,10	7,29	7,60	7,79	10,31	10,50	12,83	13,02	15,35	15,54	15,98	16,17	16,80	16,99	21,13

CARDIOBOX Table for Canine Patients Weighing 17,5 kg. Use it exclusively with proper citation of the source in your echocardiographic report. © 2024

Source: Curra-Gagliano FJ, Engel-Manchado J, Ceballos M, Redondo I A new graphical method for displaying two-dimensional echocardiography results in dogs: Comprehensive Analysis of Results of Diagnostic Imaging Organized in a BOX (CARDIOBOX). 2024.
