# Supplementary material for: A New Graphical Method for Displaying Two-Dimensional Echocardiography Results in Dogs: Comprehensive Analysis of Results of Diagnostic Imaging Organized in a BOX (CARDIOBOX)
Source: Vet Sci. 2025 Jan 9;12(1):34. doi: 10.3390/vetsci12010034 (PMC11769013; doi:10.3390/vetsci12010034)
Supplement: Supplementary file 1 [file vetsci-12-00034-s001.zip › Table S3-cardiobox table 20-50kg.pdf]

WEIGHT (kg)

20,00

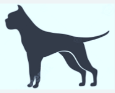

parameter	A	B	C	D	E	F	G	H	I	J	K	L	M	N	O	P	Q	R
							min					max						
Aid	9,72	11,59	11,87	13,46	13,74	15,33	15,61	19,89	20,17	24,17	24,46	28,46	28,74	35,44	35,72	42,70	42,98	62,44
IVSd	4,57	4,94	5,14	5,30	5,51	5,67	5,87	7,88	8,09	9,89	10,10	11,90	12,11	12,54	12,74	13,38	13,59	17,29
LVDd	16,43	19,80	20,05	23,18	23,42	26,55	26,79	31,50	31,74	36,20	36,45	40,91	41,15	50,01	50,25	59,35	59,59	73,23
PLVWd	3,72	4,17	4,37	4,61	4,81	5,05	5,25	7,40	7,60	9,55	9,75	11,70	11,90	12,26	12,46	13,02	13,22	17,14
IVSs	2,34	4,39	4,60	6,44	6,65	8,50	8,70	11,39	11,59	14,07	14,28	16,76	16,97	17,51	17,72	18,47	18,68	25,00
LVDs	6,15	7,84	8,10	9,52	9,78	11,21	11,47	16,65	16,90	21,82	22,08	27,00	27,26	33,67	33,93	40,60	40,85	65,57
PLVWs	6,29	6,80	7,00	7,31	7,51	7,83	8,02	10,62	10,81	13,22	13,41	15,81	16,01	16,46	16,66	17,31	17,50	21,77

CARDIOBOX Table for Canine Patients Weighing 20 kg. Use it exclusively with proper citation of the source in your echocardiographic report. © 2024

Source: Curra-Gagliano FJ, Engel-Manchado J, Ceballos M, Redondo I A new graphical method for displaying two-dimensional echocardiography results in dogs: Comprehensive Analysis of Results of Diagnostic Imaging Organized in a BOX (CARDIOBOX). 2024.

WEIGHT (kg)

22,50

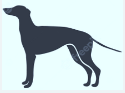

parameter	A	B	C	D	E	F	G	H	I	J	K	L	M	N	O	P	Q	R
							min					max						
Aid	10,12	12,07	12,36	14,02	14,31	15,97	16,26	20,72	21,01	25,18	25,47	29,64	29,93	36,91	37,20	44,47	44,77	65,03
IVSd	4,70	5,08	5,29	5,45	5,67	5,83	6,04	8,11	8,32	10,18	10,39	12,24	12,46	12,90	13,11	13,77	13,98	17,78
LVDd	17,01	20,50	20,75	23,99	24,24	27,49	27,74	32,61	32,86	37,48	37,73	42,35	42,60	51,77	52,02	61,44	61,69	75,82
PLVWd	3,83	4,28	4,49	4,74	4,94	5,19	5,40	7,61	7,81	9,81	10,02	12,02	12,23	12,60	12,81	13,39	13,59	17,61
IVSs	2,41	4,52	4,73	6,63	6,84	8,74	8,95	11,71	11,93	14,48	14,69	17,24	17,45	18,02	18,23	19,00	19,21	25,72
LVDs	6,38	8,13	8,40	9,88	10,15	11,64	11,90	17,27	17,54	22,65	22,91	28,02	28,29	34,94	35,21	42,13	42,40	68,05
PLVWs	6,45	6,98	7,18	7,51	7,71	8,04	8,24	10,90	11,10	13,57	13,76	16,23	16,43	16,90	17,10	17,77	17,96	22,34

CARDIOBOX Table for Canine Patients Weighing 22,5 kg. Use it exclusively with proper citation of the source in your echocardiographic report. © 2024

Source: Curra-Gagliano FJ, Engel-Manchado J, Ceballos M, Redondo I A new graphical method for displaying two-dimensional echocardiography results in dogs: Comprehensive Analysis of Results of Diagnostic Imaging Organized in a BOX (CARDIOBOX). 2024.

WEIGHT (kg)

25,00

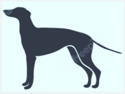

parameter	A	B	C	D	E	F	G	H	I	J	K	L	M	N	O	P	Q	R
							min					max						
Aid	10,49	12,52	12,82	14,54	14,84	16,56	16,86	21,49	21,79	26,11	26,41	30,73	31,04	38,28	38,58	46,12	46,42	67,44
IVSd	4,82	5,21	5,43	5,59	5,81	5,98	6,20	8,32	8,53	10,44	10,65	12,56	12,78	13,23	13,45	14,12	14,34	18,24
LVDd	17,54	21,15	21,40	24,75	25,01	28,35	28,61	33,63	33,89	38,66	38,92	43,68	43,94	53,40	53,66	63,38	63,63	78,20
PLVWd	3,92	4,39	4,60	4,86	5,07	5,32	5,53	7,79	8,01	10,06	10,27	12,32	12,53	12,91	13,12	13,72	13,93	18,05
IVSs	2,47	4,63	4,85	6,80	7,02	8,96	9,18	12,01	12,23	14,85	15,07	17,68	17,90	18,48	18,69	19,49	19,70	26,38
LVDs	6,60	8,41	8,69	10,22	10,49	12,03	12,30	17,86	18,13	23,41	23,69	28,97	29,24	36,12	36,40	43,55	43,83	70,34
PLVWs	6,61	7,15	7,35	7,69	7,89	8,23	8,43	11,16	11,36	13,89	14,09	16,61	16,82	17,30	17,50	18,19	18,39	22,87

CARDIOBOX Table for Canine Patients Weighing 25 kg. Use it exclusively with proper citation of the source in your echocardiographic report. © 2024

Source: Curra-Gagliano FJ, Engel-Manchado J, Ceballos M, Redondo I A new graphical method for displaying two-dimensional echocardiography results in dogs: Comprehensive Analysis of Results of Diagnostic Imaging Organized in a BOX (CARDIOBOX). 2024.

WEIGHT (kg)

30,00

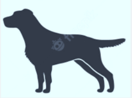

parameter	A	B	C	D	E	F	G	H	I	J	K	L	M	N	O	P	Q	R
							min					max						
Aid	11,18	13,33	13,65	15,48	15,80	17,63	17,96	22,88	23,20	27,80	28,13	32,73	33,05	40,76	41,08	49,12	49,44	71,82
IVSd	5,04	5,44	5,67	5,84	6,07	6,25	6,47	8,69	8,92	10,91	11,13	13,12	13,35	13,82	14,05	14,75	14,98	19,06
LVDd	18,51	22,31	22,58	26,11	26,38	29,91	30,18	35,49	35,76	40,79	41,06	46,09	46,36	56,34	56,61	66,87	67,14	82,51
PLVWd	4,09	4,58	4,80	5,06	5,29	5,55	5,77	8,13	8,35	10,49	10,71	12,85	13,07	13,47	13,69	14,31	14,53	18,83
IVSs	2,58	4,84	5,07	7,10	7,33	9,36	9,59	12,55	12,78	15,51	15,74	18,47	18,70	19,30	19,53	20,36	20,58	27,56
LVDs	6,99	8,91	9,20	10,82	11,11	12,74	13,03	18,91	19,21	24,80	25,09	30,68	30,97	38,26	38,55	46,13	46,42	74,50
PLVWs	6,88	7,44	7,65	8,00	8,22	8,57	8,78	11,62	11,83	14,46	14,67	17,30	17,51	18,01	18,23	18,94	19,15	23,82

CARDIOBOX Table for Canine Patients Weighing 30 kg. Use it exclusively with proper citation of the source in your echocardiographic report. © 2024

Source: Curra-Gagliano FJ, Engel-Manchado J, Ceballos M, Redondo I A new graphical method for displaying two-dimensional echocardiography results in dogs: Comprehensive Analysis of Results of Diagnostic Imaging Organized in a BOX (CARDIOBOX). 2024.

WEIGHT (kg)

35,00

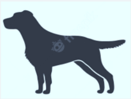

parameter	A	B	C	D	E	F	G	H	I	J	K	L	M	N	O	P	Q	R
							min					max						
Aid	11,79	14,06	14,40	16,33	16,67	18,60	18,94	24,13	24,47	29,32	29,66	34,52	34,86	42,99	43,33	51,80	52,14	75,74
IVSd	5,23	5,65	5,88	6,07	6,30	6,48	6,72	9,03	9,26	11,32	11,55	13,62	13,85	14,35	14,58	15,31	15,55	19,78
LVDd	19,37	23,35	23,63	27,32	27,61	31,30	31,58	37,13	37,42	42,68	42,96	48,23	48,51	58,95	59,24	69,97	70,25	86,33
PLVWd	4,24	4,74	4,97	5,25	5,48	5,75	5,98	8,43	8,66	10,87	11,10	13,32	13,55	13,96	14,19	14,83	15,06	19,51
IVSs	2,68	5,02	5,26	7,37	7,61	9,72	9,95	13,03	13,26	16,10	16,33	19,17	19,41	20,03	20,27	21,13	21,36	28,59
LVDs	7,34	9,35	9,66	11,36	11,67	13,37	13,68	19,85	20,16	26,03	26,34	32,21	32,51	40,16	40,47	48,42	48,73	78,21
PLVWs	7,12	7,70	7,92	8,28	8,50	8,86	9,08	12,02	12,24	14,96	15,18	17,90	18,12	18,64	18,86	19,60	19,82	24,65

CARDIOBOX Table for Canine Patients Weighing 35 kg. Use it exclusively with proper citation of the source in your echocardiographic report. © 2024

Source: Curra-Gagliano FJ, Engel-Manchado J, Ceballos M, Redondo I A new graphical method for displaying two-dimensional echocardiography results in dogs: Comprehensive Analysis of Results of Diagnostic Imaging Organized in a BOX (CARDIOBOX). 2024.

WEIGHT (kg)

40,00

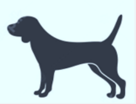

parameter	A	B	C	D	E	F	G	H	I	J	K	L	M	N	O	P	Q	R
							min					max						
Aid	12,34	14,72	15,08	17,10	17,45	19,47	19,83	25,27	25,62	30,71	31,06	36,14	36,50	45,01	45,37	54,24	54,60	79,31
IVSd	5,40	5,83	6,08	6,26	6,51	6,70	6,94	9,31	9,56	11,69	11,93	14,06	14,31	14,82	15,06	15,81	16,06	20,43
LVDd	20,14	24,28	24,58	28,42	28,71	32,55	32,85	38,62	38,91	44,39	44,68	50,16	50,45	61,31	61,61	72,77	73,06	89,79
PLVWd	4,37	4,89	5,13	5,41	5,65	5,93	6,17	8,69	8,93	11,21	11,45	13,74	13,97	14,40	14,63	15,30	15,53	20,12
IVSs	2,76	5,19	5,43	7,61	7,85	10,03	10,28	13,45	13,69	16,62	16,86	19,80	20,04	20,68	20,93	21,81	22,06	29,52
LVDs	7,65	9,75	10,07	11,85	12,17	13,95	14,27	20,71	21,03	27,15	27,47	33,59	33,91	41,89	42,21	50,50	50,82	81,57
PLVWs	7,33	7,93	8,16	8,53	8,76	9,13	9,36	12,39	12,61	15,41	15,64	18,44	18,67	19,20	19,43	20,19	20,41	25,39

CARDIOBOX Table for Canine Patients Weighing 40 kg. Use it exclusively with proper citation of the source in your echocardiographic report. © 2024

Source: Curra-Gagliano FJ, Engel-Manchado J, Ceballos M, Redondo I A new graphical method for displaying two-dimensional echocardiography results in dogs: Comprehensive Analysis of Results of Diagnostic Imaging Organized in a BOX (CARDIOBOX). 2024.

WEIGHT (kg)

45,00

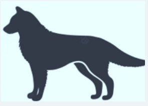

parameter	A	B	C	D	E	F	G	H	I	J	K	L	M	N	O	P	Q	R
							min					max						
Aid	12,85	15,33	15,70	17,80	18,18	20,28	20,65	26,32	26,69	31,98	32,35	37,64	38,02	46,88	47,25	56,49	56,86	82,60
IVSd	5,56	6,00	6,25	6,44	6,70	6,89	7,14	9,58	9,83	12,03	12,28	14,47	14,72	15,24	15,49	16,27	16,52	21,02
LVDd	20,85	25,14	25,44	29,42	29,72	33,70	34,01	39,98	40,28	45,95	46,26	51,93	52,23	63,48	63,78	75,33	75,64	92,95
PLVWd	4,50	5,03	5,27	5,56	5,81	6,10	6,34	8,93	9,17	11,52	11,77	14,12	14,36	14,80	15,04	15,72	15,96	20,68
IVSs	2,84	5,34	5,59	7,83	8,08	10,32	10,57	13,84	14,08	17,10	17,35	20,36	20,61	21,28	21,53	22,44	22,69	30,37
LVDs	7,94	10,12	10,45	12,30	12,63	14,47	14,81	21,49	21,82	28,17	28,51	34,86	35,19	43,47	43,80	52,41	52,74	84,65
PLVWs	7,53	8,14	8,38	8,76	8,99	9,37	9,60	12,71	12,95	15,82	16,05	18,93	19,16	19,71	19,94	20,72	20,95	26,06

CARDIOBOX Table for Canine Patients Weighing 45 kg. Use it exclusively with proper citation of the source in your echocardiographic report. © 2024

Source: Curra-Gagliano FJ, Engel-Manchado J, Ceballos M, Redondo I A new graphical method for displaying two-dimensional echocardiography results in dogs: Comprehensive Analysis of Results of Diagnostic Imaging Organized in a BOX (CARDIOBOX). 2024.

WEIGHT (kg)

50,00

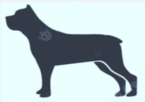

parameter	A	B	C	D	E	F	G	H	I	J	K	L	M	N	O	P	Q	R
							min					max						
Aid	13,33	15,90	16,28	18,46	18,85	21,03	21,42	27,29	27,67	33,16	33,55	39,04	39,42	48,62	49,00	58,58	58,97	85,66
IVSd	5,70	6,16	6,41	6,61	6,87	7,07	7,32	9,83	10,09	12,34	12,59	14,84	15,10	15,64	15,89	16,69	16,94	21,56
LVDd	21,51	25,93	26,24	30,34	30,66	34,76	35,08	41,24	41,55	47,40	47,71	53,56	53,87	65,47	65,79	77,70	78,02	95,88
PLVWd	4,61	5,15	5,40	5,70	5,95	6,25	6,50	9,15	9,40	11,81	12,06	14,47	14,71	15,16	15,41	16,11	16,36	21,19
IVSs	2,92	5,47	5,73	8,03	8,29	10,59	10,84	14,19	14,45	17,54	17,79	20,88	21,14	21,82	22,08	23,01	23,27	31,15
LVDs	8,21	10,46	10,80	12,71	13,05	14,96	15,31	22,22	22,56	29,13	29,47	36,04	36,38	44,94	45,28	54,18	54,52	87,51
PLVWs	7,71	8,34	8,57	8,96	9,20	9,59	9,83	13,01	13,25	16,20	16,43	19,38	19,62	20,18	20,41	21,21	21,45	26,68

CARDIOBOX Table for Canine Patients Weighing 50 kg. Use it exclusively with proper citation of the source in your echocardiographic report. © 2024

Source: Curra-Gagliano FJ, Engel-Manchado J, Ceballos M, Redondo I A new graphical method for displaying two-dimensional echocardiography results in dogs: Comprehensive Analysis of Results of Diagnostic Imaging Organized in a BOX (CARDIOBOX). 2024.
